# Value assessment of artificial intelligence in medical imaging: a scoping review

**DOI:** 10.1186/s12880-022-00918-y

**Published:** 2022-10-31

**Authors:** Iben Fasterholdt, Mohammad Naghavi-Behzad, Benjamin S. B. Rasmussen, Tue Kjølhede, Mette Maria Skjøth, Malene Grubbe Hildebrandt, Kristian Kidholm

**Affiliations:** 1 CIMT – Centre for Innovative Medical Technology, Odense University Hospital, Sdr. Boulevard 29, Entrance 102, 4rd Floor, 5000 Odense C, Denmark; 2 Department of Clinical Research, University of Southern Denmark, Odense, Denmark; 3 Department of Nuclear Medicine, Odense University Hospital, Odense, Denmark; 4 Department of Radiology, Odense University Hospital, Odense, Denmark; 5 Department of Dermatology and Allergy Centre, Odense University Hospital, Odense, Denmark; 6 CAI-X – Centre for Clinical Artificial Intelligence, Odense University Hospital, Odense, Denmark

**Keywords:** Scoping review, Value assessment, Evaluation, Artificial intelligence, Medical imaging

## Abstract

**Background:**

Artificial intelligence (AI) is seen as one of the major disrupting forces in the future healthcare system. However, the assessment of the value of these new technologies is still unclear, and no agreed international health technology assessment-based guideline exists. This study provides an overview of the available literature in the value assessment of AI in the field of medical imaging.

**Methods:**

We performed a systematic scoping review of published studies between January 2016 and September 2020 using 10 databases (Medline, Scopus, ProQuest, Google Scholar, and six related databases of grey literature). Information about the context (country, clinical area, and type of study) and mentioned domains with specific outcomes and items were extracted. An existing domain classification, from a European assessment framework, was used as a point of departure, and extracted data were grouped into domains and content analysis of data was performed covering predetermined themes.

**Results:**

Seventy-nine studies were included out of 5890 identified articles. An additional seven studies were identified by searching reference lists, and the analysis was performed on 86 included studies. Eleven domains were identified: (1) health problem and current use of technology, (2) technology aspects, (3) safety assessment, (4) clinical effectiveness, (5) economics, (6) ethical analysis, (7) organisational aspects, (8) patients and social aspects, (9) legal aspects, (10) development of AI algorithm, performance metrics and validation, and (11) other aspects. The frequency of mentioning a domain varied from 20 to 78% within the included papers. Only 15/86 studies were actual assessments of AI technologies. The majority of data were statements from reviews or papers voicing future needs or challenges of AI research, i.e. not actual outcomes of evaluations.

**Conclusions:**

This review regarding value assessment of AI in medical imaging yielded 86 studies including 11 identified domains. The domain classification based on European assessment framework proved useful and current analysis added one new domain. Included studies had a broad range of essential domains about addressing AI technologies highlighting the importance of domains related to legal and ethical aspects.

**Supplementary Information:**

The online version contains supplementary material available at 10.1186/s12880-022-00918-y.

## Background

Artificial Intelligence (AI) includes various technologies based on advanced algorithms and learning systems. Different terms are used in connection with AI, such as machine learning, deep learning, and conventional neural networks [1]. Furthermore, there is no universally agreed-upon definition of AI, while it is suggested to define it as a system capable of interpreting and learning from data to produce a specific goal [2].

AI is seen as a digital transformation and could be one of the major disrupting tools in the future healthcare system [3]. Radiology and other imaging areas encompass a vast amount of manual image reviews and a steep increase in medical images has been observed in the last decade, which requires more interpretation times by the imaging specialist. This limiting factor could be reduced by incorporating computer-aided algorithms of machine learning into the clinical workflow [4]. Pattern recognition in medical images and AI technologies seems to be a good match [5], and this area is likely to be one of the first to benefit from AI, which provides high expectations. In the first quarter of 2019, funding in imaging AI companies exceeded 1.2 billion USD [6]. A consultancy company values the annual market for the top 10 AI-based healthcare solutions at 150 billion USD in 2026 [7]. Health care payers' and providers' expectations are to achieve cost savings, improve patient satisfaction, and optimise workforce resources [8].

Most of the published AI studies within medical imaging are retrospective with a technical focus, including reporting of clinical performance metrics, validation, or robustness of the model [9]. Evaluation of this phase is thoroughly described, e.g. in the guideline Checklist for Artificial Intelligence in Medical Imaging—CLAIM [10]. However, the lack of proven clinical utility, feasibility and effect on patient outcomes has been mentioned by several studies [11–14] as well as ethical, legal, economic, sharing of data, and implementation issues [15–17]. AI is a complex technology and implementing it in a complex healthcare system, critical to society, requires a broad framework.

Health technology assessment (HTA) provides a broad framework for valuing healthcare technologies and with several examples of being tailored for specific areas like telemedicine [18] and digital healthcare services [19]. Value is to be understood in a broad sense as referring to impact or effect in several different domains. HTA is a multidisciplinary process that summarizes information that has been collected in a systematic, transparent, unbiased, and robust manner. One example is the HTA-framework from EUnetHTA [20], where evaluation is performed from nine perspectives called “domains”: (1) the health problem and current use of technology; (2) description and technical characteristics of the new technology; (3) safety assessment; (4) clinical effectiveness; (5) economic evaluation; (6) ethical analysis; (7) organisational aspects; (8) patient and social aspects; and (9) legal aspects.

It is quite important to develop a holistic and tailored HTA tool for evaluating the value of AI to implement the correct AI technologies in the field of medical imaging. Therefore, we aimed to give a comprehensive overview of relevant identified domains in the literature when assessing the value of AI in medical imaging. This is the first step in creating an evidence-based assessment tool for valuing AI technology.

## Methods

A scoping review aims to ‘map the key concepts underpinning a research area and the main sources and types of evidence available’ [21]. As such, scoping reviews typically address broad questions, potentially include a range of methodologies and do not undertake quality assessment. This contrasts with the focused question of a systematic review, which is answered from a relatively narrow range of quality-assessed studies. This scoping review was conducted based on the PRSIMA guideline [22]. The study conducted at the Centre for Innovative Medical Technology at Odense University Hospital (Denmark), covering studies between January 2016 and September 2020, including five following stages.

### Stage 1: Identifying the research issues

All published papers containing information about assessment of value of AI-technology in the field of medical imaging within public healthcare organisations, e.g. hospitals, dentists and universities, are considered eligible. Three types of studies are included to cover all aspects of AI in medical imaging: (1) Actual evaluation of the value of a specific AI technology, (2) Guidelines, statements, recommendations, white papers, evaluation models or HTA frameworks, and (3) Review articles and surveys voicing future needs/research/challenges. Hence, we reviewed available literature related to the assessment of the value of AI in medical imaging for a comprehensive understanding of which HTA domains or topics are used—or perceived relevant.

### Stages 2 and 3: Identifying relevant studies and study selection

The search terms are summarised in Table 1. Clusters one and two are inspired by a previous review by Elhakim et al. [23] and cluster three by a search strategy used in a model for telemedicine [24]. The search strategy was verified by an experienced librarian from the University of Southern Denmark. Duplications were removed and most of the article selection process was conducted in Covidence tool [25].

**Table 1:** Search terms for performing a literature review

	MeSH terms	Free text terms	
**Cluster 1: Technology**	Artificial intelligence	Artificial intelligence	
		Machine learning	
		Deep learning	
**Cluster 2: Clinical area**	Diagnostic imaging	Diagnostic imaging	
		Biomedical imaging	
		Medical imaging	
**Cluster 3: Framework (and specific domains)**	Delivery of health care	*BROAD APPROACH:*	*FOCUS ON HEA & ORGANISATIONAL ISSUES:*
	Guidelines as topic	Checklist*	
	Outcome and process assessment, Health care	Guide*	Health economic
	Program evaluation	Framework*	Cost effectiveness
	Technology assessment, Biomedical	HTA”	Cost utility analys*
	Economics	Health Technology Assessment*	Organizational
	Models, Organizational*	Assessment*	
	Ethics	Evaluation Model*	

The search string was executed as [(Cluster 1) AND (cluster 2) AND (cluster 3)]

*MeSH* Medical Subject Headings

Literature searches was conducted during 17–18th September 2020 on following 10 databases: (1) Medline (Ovid), (2) Scopus, (3) ProQuest (includes EconLit), (4) Google Scholar, and six related grey literature and working paper resources, i.e. (5) International HTA Database [26], (6) OpenGrey [27], (7) National Institutes of Health [28], (8) National Health Services [29], (9) Folkehelseinstituttet [30], 10) Folkhälsomyndigheten [31]. A single backward snowballing was done on the 1–2 most central studies selected by each of the extractors to identify further studies. Developing the selection criteria was an iterative process, conducted by the entire research group through multiple consensus meetings. Two researchers (IF and TK) performed the abstract and the full-text screenings, independently, solving the disagreements mainly by supervision of the senior researcher (KK). The inclusion and exclusion criteria are shown in Table 2.

**Table 2:** Inclusion and exclusion criteria for selection of studies

**Inclusion criteria**	1. Included studies must cover domains/topics used—or mentioned as useful—for assessment of the value of AI in the area of medical imaging:
	a. Actual evaluation or assessment of the value of a specific AI technology, i.e. AI interpretation, classification or pattern recognition of an image
	b. Guidelines, statements, recommendations, white papers, evaluation models or (HTA) frameworks described in the literature and used for assessing the value of AI
	c. Reviews, surveys voicing future needs/research/challenges and future work when evaluating the value of AI
	2. Studies must be published in English between January 1st 2016 and September 18th 2020
	3. Setting: Articles written within a hospital setting or public healthcare organisations are included, e.g., hospitals, dentists, universities, etc
**Exclusion criteria**	4. Type of study or publication: All studies including grey literature, reports, and books but we exclude citations, patents, conference book, book of collected congress contributions, and conference abstracts
	a. Because of many hits at the full-text stage, we later excluded: opinion, commentary, or viewpoint articles
	5. Studies only reporting on clinical efficacy or performance metrics, validation studies, technical development of the prediction model or AI-model, i.e., studies focusing on reporting on, sensitivity, specificity, diagnostic accuracy, precision, AUC, software used, the robustness of the model, etc
	a. Technical interpretation of images, including image optimisation and enhancing methods for highlighting images
	6. AI on non-clinical images like images of surgical equipment, plants and animals or not on images, e.g. AI on electronic health records or workflows, AI in drug development, surgical robotics, electrocardiogram, thermographic scans, brachytherapy treatment, or augmented reality visualization and VR

### Stages 4 and 5: Charting the data and collating, summarising, and reporting the results

Initially, the required information on the included studies was extracted and summarised using an extraction template in Microsoft Office Excel software. Included studies were reviewed by two researchers independently. The extracted items were: Context (country, clinical area(s), type of study); mentioned domains in the study, including specific outcomes and items in each domain. The domain classification from EUnetHTA was used as a point of departure to group the extracted data into nine domains. If a topic did not fit these pre-existing domains, the extracted text was noted in the section of “other aspects” for later evaluation. Regarding the domains “clinical effectiveness” and “the health problem and current use of technology”, specific clinical outcomes are not part of the data extraction in this scoping review. We identified a new domain (development of AI algorithm, performance metrics and validation) but did not extract specific data as this domain has been thoroughly described already. For quality assurance purposes, the principal investigator (IF) had several bilateral consultations and group sessions with all extractors to solve any inconsistencies and challenges. Accordingly, a common understanding and agreement about which topics should go under which domains, how detailed or deep to extract data, arguments for excluded studies, was reached and helped align the raw data.

In the second phase, extracted data was analysed (by two researchers) and condensed for each domain. Qualitative content analysis was performed [32], covering four predetermined themes/questions: (1) Effects, outcomes, value or impacts mentioned—as well as future needs/challenges/topics relevant when evaluating the value of AI, (2) Specific outcome measures, (3) Frequency of using a given topic or outcome group, (4) Potential overlap with other domains. A summary for each domain was made as the last step of analysis.

## Results

**Fig. 1:**
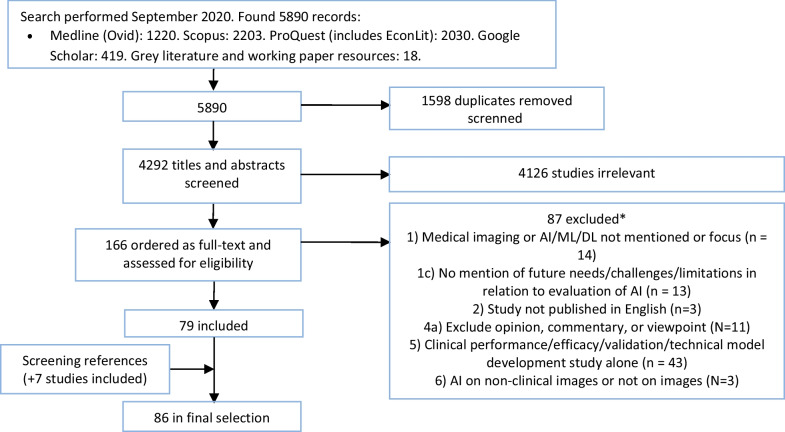
PRISMA flow chart for selection of the studies. *The number in front of the list with exclusion reasons refers to the exclusion criteria in Table 2

A study flow chart summarizes the process of literature retrieval (Fig. 1). In total, the literature search yielded 5890 papers, while 4292 papers remained after the removal of duplicates.

Based on titles and abstracts 166 papers were eligible for the full-text assessment, and a total of 79 papers [11, 12, 19, 33–108] fulfilled the inclusion criteria (see Additional file 1). Further, seven papers were included based on screening of reference lists in the most central articles [109–115]. Hence, 86 papers were included in the scoping review.

### Characteristics and analysis of included AI-studies

The characteristics of the 86 included studies are presented in Table 3. The most frequent clinical areas covered are radiology (17%), medical imaging (14%) and radiomics (13%). Most studies (n = 61) are voicing future needs or challenges when evaluating AI, while there were also studies containing an actual evaluation (n = 15) as well as guideline, statement or framework (n = 10). Most studies are published in 2019 and 2020. The most frequently mentioned or perceived relevant domains are the development of AI algorithm, performance metrics and validation (78%), followed by technology aspects (73%). Excluding “other aspects”, the variation between how often domains is mentioned is in the range of 20% to 78%.

**Table 3:** Characteristics of included studies (N = 86)

Element in study	Categories	Frequency (%)
Country of 1.author	Australia	3 (3%)
	Asia and the Middle East (China, Hong Kong, India, Israel, Japan, Saudi Arabia, Singapore, South Korea)	16 (19%)
	Canada	6 (7%)
	Europe (Cyprus, Denmark, Finland, France, Germany, Italy, Netherland, Portugal, Spain, Sweden)	23 (27%)
	UK	8 (9%)
	US	26 (30%)
	Unclear	1 (1%)
	Other (Norway, Switzerland)	3 (3%)
Clinical area covered	Breast cancer (mammography and digital breast tomosynthesis)	9 (10%)
	Dementia/Alzheimer´s disease/neuroimaging	7 (8%)
	Dermatology (melanoma diagnosis, histopathologic images)	2 (2%)
	Cardiovascular disease (cardiovascular imaging, coronary artery disease)	8 (9%)
	Diabetes and ophthalmology (ocular imaging, diabetic retinopathy screening, diabetic eye disease screening)	6 (7%)
	Oncology and radiotherapy	8 (9%)
	Radiology	15 (17%)
	Radiomics	11 (13%)
	Medical imaging	12 (14%)
	Pathology (histopathology images)	2 (2%)
	Other	6 (7%)
Study type	Actual evaluation	15 (17%)
	Guidelines, statements or frameworks	10 (12%)
	Reviews, surveys or papers voicing future needs or challenges	61 (71%)
Year of publication	2016	0 (0%)
	2017	11 (13%)
	2018	9 (10%)
	2019	37 (43%)
	2020 (mid-September)	29 (34%)
Domain mentioned or perceived relevant	1. The health problem and current use of technology	55 (64%)
	2. Technology aspects	63 (73%)
	3. Safety assessment	17 (20%)
	4. Clinical effectiveness, e.g. clinical outcomes	39 (45%)
	5. Economics	52 (60%)
	6. Ethical analysis	25 (29%)
	7. Organisational aspects	53 (62%)
	8. Patients and social aspects	33 (38%)
	9. Legal aspects	43 (50%)
	10. Development of AI algorithm, performance metrics and validation	67 (78%)
	11. Other aspects	2 (2%)

In total, we identified eleven relevant domains about assessing the value of AI (two more than the EUnetHTA domain classification). In addition, we extracted more specific data for eight domains including technology, safety, economics, ethics, organisational, patients and social, legal, and the other aspects. Regarding the remaining three domains (clinical effectiveness, the health problem and current use of technology, and development of AI algorithm, performance metrics and validation), we only noted whether information was present or not. Table 4 contains summary and specific outcome measures (if applicable) through extracted information of the included domains. Further details can be found in Additional file 2.

**Table 4:** Summary of extracted data for each specific domain

Domain	Topics or themes and outcome measures
(1) The health problem and current use of technology	No details extracted
(2) Technology aspects	The main topic is interpretability in the sense that we need to avoid the “Black box problem” and the analysis done by the algorithm needs to be transparent to physicians/staff i.e., explainable AI
	Furthermore, risk of bias, possibly causes discrimination issues and validation. The algorithm development method is highlighted, including data quality, the importance of annotation, external evaluation, and reference standards
	Equipment and IT was a topic mentioning the clinical IT integration and infrastructure
	*OUTCOME MEASURES:* interpretability, quality of scans, technical functioning/feasibility
(3) Safety assessment	Safety of patients, potential challenges after implementation of AI to the healthcare system
	Reducing side effects and especially radiation dose, data security and protection
	*OUTCOME MEASURES*: natural radiation exposure, using clinical knowledge support
(4) Clinical effectiveness, e.g. clinical outcomes*	No details extracted
(5) Economics	The description of the savings and benefits are most often very general, e.g. improved cost-effectiveness
	*OUTCOME MEASURES*: reduction in workload and time for staff, reduction in the number of biopsies and patients use of medication
(6) Ethical analysis	Privacy, consent, obligations, security, awareness of the use of patients’ data, and ownership of the data
	Ethical approval and consider ethical issues of data, algorithms, trained models, and practice
	Understanding risks vs. benefits, shared/clear decision-making and transparency of results
	Big questions: “who owns data”, “can data and the algorithm be trusted” and “what is good clinical practice?
	*OUTCOME MEASURES:* validity of data, risks versus benefits, patient safety
(7) Organisational aspects	Benefits in the form of reductions in workflow and tasks related to imaging for the staff as a result of AI
	The use of additional time related to implementation and training and the challenges related to ensuring acceptability
	*OUTCOME MEASURES:* changes in time use for the health care professionals and patient, clinician acceptability measures
(8) Patients and social aspects	Patients’ comfortability including easier imaging process and providing access to own data/report in a safe and secured platform
	Better treatment outcome based on the improved clinical decision is the most discussed issue
	Patients’ satisfaction, as well as clinical benefits, could result in better acceptability of AI technology in the healthcare system
	*OUTCOME MEASURES:* the time required for diagnosis, rating for overall satisfaction, help patients make more informed activity choices
(9) Legal aspects	Data security and privacy
	Responsibility for misdiagnosis
	*OUTCOME MEASURES:* regulatory approvals, consent from patients
(10) Development of AI algorithm, performance metrics and validation	No details extracted
(11) Other aspects	Overpromising language in studies
	Offering the possibility of performing expensive and time-consuming screening programs in countries that otherwise cannot afford them
	*OUTCOME MEASURES:* none identified

The full data analysis shows frequent overlap to other domains [see Additional file 2]

### Clinical effects (domain 4)

Although clinical outcomes are not part of the extraction of the included studies in the scoping review the clinical domain is evidently of great importance. Several outcome measures for clinical effectiveness studies have been suggested in a systematic scoping review of AI methods applied to adult patients who underwent any health/medical intervention and reported therapeutic, preventive, or prognostic outcomes [116]. Based on 370 studies, this review found that AI was used primarily for the prediction/prognosis of more frequently reported outcomes, efficacy/effectiveness, and morbidity outcomes. Some examples of reasonably broad clinical or patient outcomes mentioned in the reviewed literature are fewer false positives in screening mammography, progression of disease and misdiagnosis, avoiding unnecessary stereotactic biopsies, improvement of treatment appropriateness (by physicians) and adherence (by patients) and the prevention of iatrogenic adverse events.

## Discussion

### Main findings

This scoping review regarding the value assessment of artificial intelligence in medical imaging yielded 86 included papers. Eleven domains were identified. The frequency of mentioning a domain varied from 20 to 78% within included studies. The studies were divided into three study types: studies voicing future needs or challenges when evaluating the value of AI, actual evaluations, and lastly, guidelines, statements, or frameworks. Out of 86 studies, only 15 were actual evaluations, and thus most data were based on statements and not actual outcomes of evaluations.

### Comparing findings to the literature

The domain classification from the EUnetHTA framework proved very useful as extracted data used all the nine pre-existing domains and identified only one new domain (this is apart from a few issues filed in an “other domain”). However, the studies of value assessment of AI in the area of medical imaging includes a broad range of important domains in contrast to other studies. For example, an interview study in nine European countries investigated the information needs of hospital managers when deciding about investments in new treatments [117]). In that study, legal, social and ethical aspects were not deemed very important, which is in contrast with our findings. Further, in telemedicine, a scoping review of empirical studies that have applied the Model for Assessment of Telemedicine (MAST) shows that clinical, patient and economic effects are the most important areas [24]. Perhaps AI is unique in that all domains seem rather important—or perhaps in a more empirical setting and when more late evaluations become available this picture will change.

### Limitations and strengths

Although the authors tried to provide a general overview regarding different aspects of value assessment for AI technology, there were some limitations. The terminology in the area of AI is still relatively immature. So although the focus was on medical imaging, we included articles in pathology and radiomics. Pathology and radiomics were not fully covered with our search terms, but this being a scoping review, we decided to keep these related studies and let the available data reflect in our analysis. Also, some of the included studies were narrative reviews that make it challenging to extract firm conclusions since there was no strong evidence to support mentioned results or claims. Another limitation was the considerable overlap between some domains where the included studies (and extractors) categorised data differently. This made it difficult to align the data analysis. We discussed overlaps in our joint meetings and selected the domain that best covered most of the included topics to handle these inconsistencies. For example, the topic of “explainable AI”/”black box” was initially extracted in both safety, technology, organisational and sometimes the other domain. Further, specific clinical outcomes are evidently of great importance when evaluating an AI technology. However, they were not part of the data extraction because most studies are very disease-specific and the number of included studies would have been unmanageable high in this review. This being a scooping review critical appraisal of the included studies were not done.

Regarding the strengths of this review, the broad coverage of areas of relevance for assessing the value of AI projects is in demand [17, 116, 118]. To the best of our knowledge, this is the first systematic scoping review about the value assessment of AI in field of medical imaging. Our search included grey literature which can be an important information source [119]. Furthermore, we have included the studies with different research methodologies to ensure the high coverage and a broad perspective in our data collection. The joint discussion sessions on topics with high overlap between the domains have likely improved the data analysis. High overlap was particularly observed in the topics of patient’s privacy (overlap between ethical, legal, and patient’s domains) and the earlier mentioned black box topic. Furthermore, most studies are published recently (after 2019) which shows a great interest in this field and reflects the newest information on the topic. Drawing a framework based on data from recent publications can strengthen the current value assessment to be considered in future evaluations in this field.

### Future directions

**Fig. 2:**
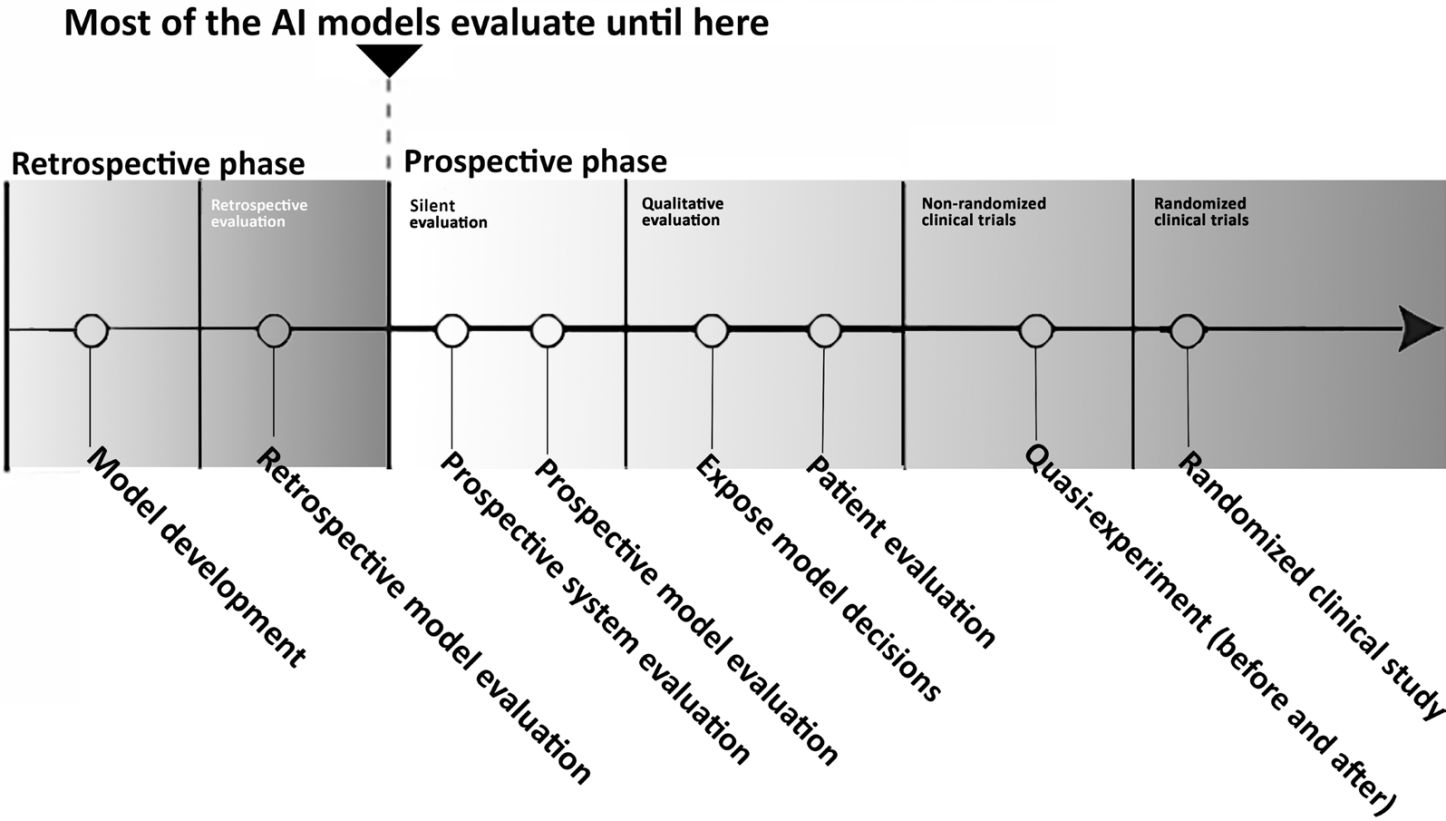
Pipeline for evaluating AI technologies (inspired by an unpublished figure by Simon Meyer Lauritsen)

A pipeline for an overview of AI technologies evaluation is shown in Fig. 2. A majority of the published AI studies within medical imaging is in the retrospective phase of Fig. 2, i.e. having a technical focus. However, as our review shows, it is important to evaluate AI projects both clinically and concerning many other areas, i.e. the prospective phase in Fig. 2, to ensure that AI technologies with no effect or unintended effects are not uncritically implemented.

Results from this review was used as part of a project with the overall aim to develop a Model for ASsessing the value of AI in medical imaging (MAS-AI) cf. publication [120]. MAS-AI was developed in three phases. First, this literature review. Next, we interviewed leading researchers in AI in Denmark. The third phase consisted of two workshops where decision-makers, patient organizations, and researchers discussed crucial topics when evaluating AI. The multidisciplinary team revised the model between workshops according to comments. The HTA framework MAS-AI is to support the introduction of AI technologies into healthcare in medical imaging.

It is important to ensure uniform and valid decisions regarding the adoption of AI technology with a structured process and tool. The MAS-AI model can help support these decisions and provide greater transparency for all parties involved.

## Conclusion

This scoping review regarding value assessment of artificial intelligence in medical imaging yielded 86 papers fulfilling the inclusion criteria, and eleven domains were identified: (1) the health problem and current use of technology, (2) technology aspects, (3) safety assessment, (4) clinical effectiveness, (5) economics, (6) ethical analysis, (7) organisational aspects, (8) patients and social aspects, (9) legal aspects, (10) development of AI algorithm, performance metrics and validation, and (11) other aspects. The domain classification from the EUnetHTA framework proved very useful and analysis identified only one new real domain: domain 10 (a few issues were included in an “other domain”). Studies include a broad range of essential domains when addressing AI technologies; in contrast to other areas, legal and ethical aspects are highlighted as important in this review.

## Supplementary Information

**Additional file 1.** Final searches for the scoping review. The final searches for the scoping review is provided.

**Additional file 2.** Full data analysis for all domains. The full data analysis for all domains is provided.

## Data Availability

The raw datasets used during the current study are available from the corresponding author on reasonable request.

## Abbreviations

AI: Artificial intelligence
CLAIM: Checklist for Artificial Intelligence in Medical Imaging
HTA: Health technology assessment
EUnetHTA: European Network for Health Technology Assessment
MAS-AI: Model for ASsessing the value of AI in medical imaging
MAST: Model for ASessment of Telemedicine
