# Correction: Value assessment of artificial intelligence in medical imaging: a scoping review

**DOI:** 10.1186/s12880-023-00965-z

**Published:** 2023-01-23

**Authors:** Iben Fasterholdt, Mohammad Naghavi-Behzad, Benjamin S. B. Rasmussen, Tue Kjølhede, Mette Maria Skjøth, Malene Grubbe Hildebrandt, Kristian Kidholm

**Affiliations:** 1 CIMT – Centre for Innovative Medical Technology, Odense University Hospital, Sdr. Boulevard 29, Entrance 102, 4rd Floor, 5000 Odense C, Denmark; 2 Department of Clinical Research, University of Southern Denmark, Odense, Denmark; 3 Department of Nuclear Medicine, Odense University Hospital, Odense, Denmark; 4 Department of Radiology, Odense University Hospital, Odense, Denmark; 5 Department of Dermatology and Allergy Centre, Odense University Hospital, Odense, Denmark; 6 CAI-X – Centre for Clinical Artificial Intelligence, Odense University Hospital, Odense, Denmark

**Correction: BMC Medical Imaging (2022) 22:187** 10.1186/s12880-022-00918-y

Following the publication of the original article [1], it was brought to our attention that an error had been introduced during typesetting:

The incorrect word “screnned” was inadvertently inserted under “1598 duplicates removed” in Fig. 1.

The incorrect word has now been deleted and the correct Fig. 1 is included in this Correction.

**Fig. 1:**
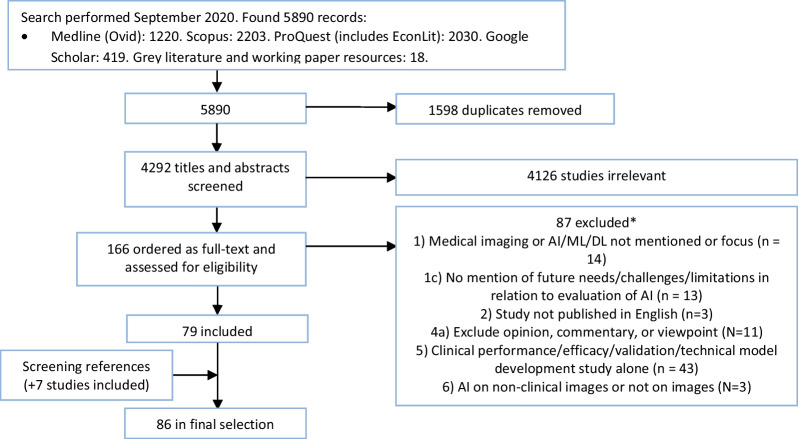
PRISMA flow chart for selection of the studies. *The number in front of the list with exclusion reasons refers to the exclusion criteria in Table 2

The original article has been updated.
