# Supplementary material for: Value assessment of artificial intelligence in medical imaging: a scoping review
Source: BMC Med Imaging. 2022 Oct 31;22:187. doi: 10.1186/s12880-022-00918-y (PMC9620604; doi:10.1186/s12880-022-00918-y)
Supplement: Supplementary file 1 — Additional file 1. Final searches for the scoping review. The final searches for the scoping review is provided. [file 12880_2022_918_MOESM1_ESM.docx]

# Final searches for the scoping review

| **Database** | **Search terms** | **Results (+ date search was done)** |
| --- | --- | --- |
| **Medline** | (Artificial Intelligence/ or "Artificial intelligence".mp. or "Machine learning".mp. or "Deep learning".mp.) and (Diagnostic Imaging/ or Diagnostic Imaging.mp. or "Biomedical imaging".mp. or "medical imaging".mp.) and (Ethics/ or "Delivery of Health Care"/ or "Guidelines as Topic"/ or "Outcome and Process Assessment, Health Care"/ or "Program Evaluation"/ or "Technology Assessment, Biomedical"/ or Economics/ or "Models, Organizational/".mp. or "checklist*".mp. or "Guide*".mp. or "Framework*".mp. or HTA.mp. or "Health Technology Assessment*".mp. or "Assessment*".mp. or "Evaluation Model*".mp. or "Cost effectiveness".mp. or "Cost utility analys*".mp. or "Health economic*".mp. or "Organizational*".mp.) | **1.220**  **(18/9-2020)** |
| **Scopus** | ( TITLE-ABS-KEY ( "Artificial intelligence" OR "Machine learning" OR "Deep learning" ) ) AND ( TITLE-ABS-KEY ( "Biomedical imaging" OR "medical imaging" OR "diagnostic imaging" ) ) AND ( TITLE-ABS-KEY ( checklist* OR guide* OR framework* OR "HTA" OR "Health Technology Assessment*" OR "Evaluation Model*" OR "Cost effectiveness" OR "Cost utility analys*" OR "health economic*" OR organizational* ) OR TITLE ( assessment* ) ) AND ( LIMIT-TO ( PUBYEAR , 2020 ) OR LIMIT-TO ( PUBYEAR , 2019 ) OR LIMIT-TO ( PUBYEAR , 2018 ) OR LIMIT-TO ( PUBYEAR , 2017 ) OR LIMIT-TO ( PUBYEAR , 2016 ) ) | **2.203**  **(18/9-2020)** |
| **ProQuest (includes EconLit)** | (ab("Artificial intelligence" OR "Machine learning" OR "Deep learning") OR ti("Artificial intelligence" OR "Machine learning" OR "Deep learning") OR mainsubject("Artificial intelligence" OR "Machine learning" OR "Deep learning")) AND (ab("Biomedical imaging" OR "medical imaging" OR "diagnostic imaging") OR ti("Biomedical imaging" OR "medical imaging" OR "diagnostic imaging") OR mainsubject("Biomedical imaging" OR "medical imaging" OR "diagnostic imaging")) AND (ab(checklist* OR guide* OR framework* OR "HTA" OR "Health Technology Assessment*" OR "evaluation model" OR "Cost effectiveness" OR "Cost utility analys*" OR "health economic*" OR organizational* OR assessment*) OR ti(checklist* OR guide* OR framework* OR "HTA" OR "Health Technology Assessment*" OR "evaluation model" OR "Cost effectiveness" OR "Cost utility analys*" OR "health economic*" OR organizational* OR assessment*) OR mainsubject(checklist* OR guide* OR framework* OR "HTA" OR "Health Technology Assessment*" OR "evaluation model" OR "Cost effectiveness" OR "Cost utility analys*" OR "health economic*" OR organizational* OR assessment*)) | **2.030**  **(18/09-2020)** |
| **Google Scholar** | "Artificial Intelligence" OR "deep learning" OR “Machine learning” "medical imaging" OR "diagnostic imaging" OR” Biomedical imaging" "Health economic" OR "Health Technology Assessment" OR “HTA” | **419**  **(17/9-2020)** |
| **Open grey** | ("Artificial Intelligence" OR "deep learning" OR “Machine learning”) | **15 - none from 2016-2020** |
| **International HTA database** | ( "Artificial intelligence" OR "Machine learning" OR "Deep learning” mh]) | **3 - none from 2016-2020** |
| **NIH, NHS, Folkehelseinstituttet, Folkhälsomyndigheten** |  | **0** |
