# Supplementary material for: Value assessment of artificial intelligence in medical imaging: a scoping review
Source: BMC Med Imaging. 2022 Oct 31;22:187. doi: 10.1186/s12880-022-00918-y (PMC9620604; doi:10.1186/s12880-022-00918-y)
Supplement: Supplementary file 2 — Additional file 2. Full data analysis for all domains. The full data analysis for all domains is provided. [file 12880_2022_918_MOESM2_ESM.docx]

# Full data analysis for all domains

Signature explanation: An "R" followed by a number refer to statements coming from an article from the literature review.

| **Domain** | **Extractor** | **Reviewer** |
| --- | --- | --- |
| **1) The health problem and current use of technology + 10) Development of AI algorithm, performance metrics and validation** | **IF** | **BSR** |

**1) Which overall effects, outcomes, value or impacts are mentioned - including future needs/challenges/topics relevant when evaluating the value of AI?**

Domain 1:

1. Item 3-4 (intended use and clinical role of the AI approach, study objectives and hypotheses)

Domain 10:

1. Item 5-32 covers methods topics:
   1. Study design
   2. Data (data sources, eligibility criteria, data preprocessing steps, de-identification methods, how missing data were handled)
   3. Ground Truth (definition of ground truth reference standard in sufficient detail to allow replication, rationale for choosing the reference standard, source of ground truth annotations + qualifications and preparation of annotators, annotation tools, measurement of inter- and intrarater variability + methods to mitigate variability and/or resolve discrepancies)
   4. Data Partitions (intended sample size and how it was determined, how data were assigned to partitions + specify proportions, level at which partitions are disjoint (eg, image, study, patient, institution))
   5. Model (Detailed description of model, including inputs, outputs, all intermediate layers and connections; software libraries, frameworks, and packages; Initialization of model parameters (eg, randomization, transfer learning)
   6. Training (details of training approach, including data augmentation, hyperparameters, number of models trained; method of selecting the final model; Ensembling techniques, if applicable)
   7. Evaluation (metrics of model performance; statistical measures of significance and uncertainty; Robustness or sensitivity analysis; Methods for explainability or interpretability (eg, saliency maps) and how they were validated; Validation or testing on external data)
2. Item 33-37 covers results topics:
   1. Data (Flow of participants or cases, using a diagram to indicate inclusion and exclusion; Demographic and clinical characteristics of cases in each partition)
   2. Model performance (performance metrics for optimal model(s) on all data partitions; Estimates of diagnostic accuracy and their precision (such as 95% confidence intervals); Failure analysis of incorrectly classified cases)

**Summary of domain**

The above data is solely based on the Checklist for Artificial Intelligence in Medical Imaging (CLAIM). The CLAIM guideline has 42 items, which are relevant for domain 1 and domain 10.

| **Domain** | **Extractor** | **Reviewer** |
| --- | --- | --- |
| **2) Technology aspects** | **BSR** | **TK** |

**1) Which overall effects, outcomes, value or impacts are mentioned - including future needs/challenges/topics relevant when evaluating the value of AI?**

1. Data quality (R20, R30,R35,R40,R42,R45,R46,R79,R20,R66,R85)
2. Interpretability/Black box: System decision interpretable or transparent to physicians/staff? (R4,R7,R21,R31,R33,R35,R37,R38,R40,R41,R48,R52,R55,R58,R60,R61,R64,R65,R66,R70,R74,R86,R90)
   1. Explainable AI (R31,R48,R52,R61,R74,R90)
   2. Transparency, e.g. open-source architecture (R40)
   3. Clinical decision-making process, separating judgments about the scan itself from the subsequent referral decision, allowing a clinician to inspect and visualize an interpretable segmentation (R41)
3. The risk of bias, possible cause discrimination issues and validation (R31,R35,R77,R79,R89,R90)
4. Validation in clinical environment before integration (R39)
5. Future recommendation/expectations (R36,R37,R43,R49,R52,R72,R79,R81,R82,R83)
   1. Reducing complexity and improving quality of scans (R83) or quality of images (R86)
   2. Convey the interpretation to clinicians in direct medical languages (R39)
   3. Provide deeper level of explanation if the recommendation is questioned (R39)
   4. Work towards a flexible, extensible, integrated framework (R40)
6. Development of model, e.g. external evaluation, validation, reference standard and quality
   1. Labeling/annotation (R8,R15)
   2. Evaluation (model) (R21,R27,R67,R79,R89)
   3. Validation of imaging and AI system to be safe (R15,R41,R63,R78,R82)
   4. Establish performance standards, quality assurance and monitoring procedures, and compliance guidance to ensure patient safety (R38,R39,R74,R87) + transform over time, i.e. meaning the AI system is improving/changing over time (R66)
   5. Eliminate unstable features, correct for influencing factors, or harmonize datasets + consider as an additional tool and not as a standalone diagnostic algorithm (R81)
   6. Quality (R13,R21,R24,R31,R40,R42,R46,R62,R66,R67,R71,R78)
7. Equipment and IT (hardware and software)
   1. Clinical IT integration, i.e. the EMR (R31,R39,R63,R69, R76)
      1. Physical localisation of computation (R69)
   2. Infrastructure (R67,R79)
   3. Computational hardware and novel neural network architectures (R75)
   4. Assess technical functioning/feasibility (R32,R88)
   5. Description of technical solution, e.g. amount of pixels, spatial resolution (R9), plugin types, GUI-integration, etc. (R11) and reducing the average computation time more than 80-fold (R80)
   6. algorithms may be developed on a single scanner type or imaging technique that is institution specific (R75, R78)

**2) How are outcomes (group 1 elements) measured, i.e. which specific outcome measures are mentioned?**

- Interpretability
- AI algorithm performance metrics and validation
  - Number of clinicians in comparator group – interobserver variability (R67,R74,R79)
- Quality of scans
- Technical functioning/feasibility

**3) How many times is a topic or outcome group mentioned?**

- Interpretability: 24
- Development of model AI algorithm, etc.: 30

**4) Describe any overlap to other domains**

Ethical (R31,R35,R77,R89,R90), safety (R35,R38,R39,R63,R81) and development of AI algorithm, performance metrics and validation performance metrics (all elements to some degree)

**Summary of domain**

The main single topic in the literature is by far interpretability in that sense that we need to avoid the “Black box problem” and the analysis done by the algorithm needs to be transparent to physicians/staff i.e., explainable AI. Next is data quality. Furthermore risk of bias, possible cause discrimination issues and validation. The development of the algorithm is highlighted including quality, the importance of annotation, external evaluation and reference standards. Furthermore, equipment and IT were a topic mentioning the clinical IT integration, and infrastructure.

| **Domain** | **Extractor** | **Reviewer** |
| --- | --- | --- |
| **3) Safety assessment** | **MNB** | **MGH** |

**1) Which overall effects, outcomes, value or impacts are mentioned - including future needs/challenges/topics relevant when evaluating the value of AI?**

1. Safety of the patients and reducing harm
2. Avoiding side effects + reducing futile expenditure (R6,R26)
3. Minimizing the harm and decreasing radiation/radiotracer dose (R9,R20,R76,R83,R86,R90)
4. Well definition of Safety and precision of AI algorithms (R34)
5. Achieving faster and safer images (R83)
6. Involving clinical trials to demonstrate safety and scientific evidence on safety (R41,R75)
7. Responsibility for safeguarding patients (R76,R77)
8. Upcoming challenges
9. Identifying all potential manners with possibility of failure, including their causes and effects (R28)
10. Considering cyberattacks, incidents (notification and minimization), and service continuity (R86)
11. The risk and benefit of training with live data and using AI tool (R48,R85)
12. Association with the underlying technology or the implementation of wider system (R56)
13. Automation bias followed by commercial uses of AI (R90)

**2) How are outcomes (group 1 elements) measured, i.e. which specific outcome measures are mentioned?**

- Comparison of natural radiation exposure (R9)
- Using ‘clinical knowledge support’ (R49)
- Methods adopted to monitor safety, e.g. regarding the issue of interpretability of artificial intelligence systems to interrogate the decision-making process (R56)
- Constantly monitoring of the risk and benefit of training with live data for unintended consequences (R85)
- Considering mechanisms to avoid patient harm in case of dealing with rare cases and generalizability (R90)

**3) How many times is a topic or outcome group mentioned?**

- Avoiding side effects + reducing futile expenditure: 2 times (R6,R26)
- Considering or decreasing radiation/radiotracer dose: 3 times (R9,R83,R86)
- Minimizing the harm: 3 times (R20,R76,R90)

**4) Describe any overlap to other domains**

The overlap could be seen between safety assessment and ethical domain regarding items about “Data protection and safety” and in relation to technology (some elements in 2)

**Summary of domain**

Safety is one of the main concerns in implementation of AI and includes concerns about safety of patients and potential challenges after implementation of AI to the healthcare system. Safety domain mostly focuses on reducing side effects and specially radiation dose, and data security and protection.

| **Domain** | **Extractor** | **Reviewer** |
| --- | --- | --- |
| **4) Clinical effectiveness, e.g. clinical outcomes** | **IF** | **BSR** |

**Summary of domain**

Clinical aspects, i.e. the clinical domain, is of great importance and 36 of the 86 studies mention clinical outcomes. However, specific clinical outcomes are not part of the data extraction from the included studies in this scoping review. The reason for this decision was that most studies are very disease specific and also the number of included studies would have been unmanageable high.

| **Domain** | **Extractor** | **Reviewer** |
| --- | --- | --- |
| **5) Economics** | **KK** | **IF** |

**1) Which overall effects, outcomes, value or impacts are mentioned - including future needs/challenges/topics relevant when evaluating the value of AI?**

1. Cost-effectiveness (R4,R7,R16,R19,R24,R26,R52,R61,R66,R80,R88,R47,R56,R82,R86)
2. Cost per QALY (quality adjusted life year) gained by use of AI (R24,R26,R80)
3. Reduced costs (R5,R19,R36,R43,R49,R76,R91,R89), reduced costs of classification of images by up to 67% (R15), reduction in number of unnecessary tests (R14,R44,R83), reduce transportation (R30), reduced patient costs (41)
4. Reduced time and work load for health professionals, e.g. for image interpretation (R8,R44,R38,R48,R54,R55,R57,R63,R67, R68,R74)
5. Change in care processes (R21)
6. Costs of screening (R7), cost of treatment (R9), cost of Device (R9)
7. Total Scan Time (R9, R10), less user time (R12)
8. Expensive upfront and ongoing costs, incl. training and implementation (R47,R56)

**2) How are outcomes (group 1 elements) measured, i.e. which specific outcome measures are mentioned?**

- number of patient investigations and number of changes in diagnosis (R6)
- time for the doctor (R8)
- costs per scan (R9)
- workload of second reader can be diminished up to 88% (R13)
- travel costs (R47)
- number of patients who need a biopsy (R48)
- time devoted to imaging interpretation decreases (R55)
- efficiency - defined as signout turnaround time - per case (R57)

**3) How many times is a topic or outcome group mentioned?**

- 58 of the 86 studies mention economic aspects of AI.
- Cost-effectiveness: 12
- Cost per QALY: 3
- Reduced costs: 19
- Reduced workload or use of time for staff: 10
- Additional cost related to investment or implementation of AI: 3

**4) Describe any overlap to other domains**

Often impact on workload and time for staff is mention, which is relevant for the organisational aspects. Also, clinical impact measures (domain 4) in QALYs.

**Summary of domain**

- Economic aspects of AI are frequently mentioned as parts of the value of AI in health care.
- In most cases descriptions of the savings and benefits are very general, e.g. improved cost-effectiveness
- The most frequent description of specific economic value of AI includes:
  - Reduction in workload and time for staff in diagnostic procedures
  - Reduction in number of biopsies and patients use of medicine as results of improved diagnostics.

| **Domain** | **Extractor** | **Reviewer** |
| --- | --- | --- |
| **6) Ethical analysis** | **MM/MGH** | **MNB/TK** |

**1) Which overall effects, outcomes, value or impacts are mentioned - including future needs/challenges/topics relevant when evaluating the value of AI?**

1. Need for ethical approval or ethics boards (R13,R26,R32,R82,R87)
2. Considering ethical issues of data, algorithms, trained models, and practice (R20,R24,R54,R68,R77,R86,R89,R90)
   1. The computer is not burdened by human limits of vision or cognition, fallibility related to stress, fatigue, hunger, learned bias or institutional pressure (R54).
3. Considering ethical issues relating to the implementation of AI (R22,R31,R52,R76,R87)
   1. Can the algorithm be trusted? Is it fair? (R31)
   2. How viable is the algorithm (R52)
4. Privacy, consent, obligations, security, awareness of use of patients’ data, and ownership of the data (R13,R23,R25,R32,R37,R45,R51,R58,R76,R82,R86,R89,R90)
   1. Who owns data (R23)
   2. How to handle data between organisations (R25)
   3. What is good clinical practice (R13,R32,R37)
   4. Can pictures of faces be 3d-reconstructed? (R90)
   5. Morality (R76), ethical principles, such as beneficence and respect for patients (R86)
5. Understanding the risks vs. benefits of sharing health information, decision making (R22,R73,R74)
   1. Transparency of results (R74)
6. Quality vs cost conflicts: If cost is factored into the algorithm, it is easy to see any number of unethical machine-generated outcomes (R85)
7. Risk of misdiagnosis, wrong treatment and potential harm to patients (R24,R48,R89)
   1. Missing cases, misdiagnosis and over over-diagnosing - for instance causing unnecessary invasive procedures such as biopsies (R89)

**2) How are outcomes (group 1 elements) measured, i.e. which specific outcome measures are mentioned?**

- Better understanding of data and the risks versus benefits of sharing health information (R22,R52)
- Establishing concrete guidelines and framework for using clinical data for research and development (R25)
- Importance of robust research and ethics board (REB) involvement in the development of AI tools in health care (R82). Having a separate review board dealing with the ethics, informed consents, data, documentation and research aspect ensuring good clinical practice (R32)
- Considering misdiagnosis and patient harmed (R89)
- Consent in use of data and choice of treatment (R23,R32)
- Using GDPR as a suitable instrument to regulate AI (R86)
- Programming AI systems to guide users toward clinical actions (R86)

**3) How many times is a topic or outcome group mentioned?**

- Need for ethical approval or ethics boards: 5 times (R13,R26,R32,R82,R87)
- Considering ethical issues of data, algorithms, trained models, and practice: 7 times (R20,R24,R68,R77,R86,R89,R90)
- Ethical issues relating to the implementation of AI: 5 times (R22,R31,R52,R76,R87)
- Privacy, consent, obligations, security, awareness of use of patients’ data, and ownership of the data: 13 times (R13,R23,R25,R32,R37,R45,R51,R58,R76,R82,R86,R89,R90)

**4) Describe any overlap to other domains**

There is a clear overlap between ethical domain and technology (R31,R35,R77,R89,R90), patient safety (R37,R48,R52) and legal aspects (R22,R89). A considerable overlap was predictable due to the nature of these domains. Ethical issues could never be solved without legal support and on the other hand rules need to be regulated based on ethical issues.

**Summary of domain**

Ethical issues are regularly brought up with respect to the use and implementation of AI. Privacy, consent, obligations, security, awareness of use of patients’ data, and ownership of the data are some of the most frequently mentioned topics. Several papers describe the need for ethical approval and highlights the importance of considering ethical issues of data, algorithms, trained models, and practice with respect to AI. More describe the importance of understanding of the risks vs. benefits, shared/clear decision making and transparency of results to avoid risk of misdiagnosis, wrong treatment and potential harm to patients. Some of the big questions is “who owns data”, “can data and the algorithm be trusted” and “what is good clinical practice”.

| **Domain** | **Extractor** | **Reviewer** |
| --- | --- | --- |
| **7) Organisational aspects** | **IF** | **KK** |

**1) Which overall effects, outcomes, value or impacts are mentioned - including future needs/challenges/topics relevant when evaluating the value of AI?**

1. Changes in workflow: Saving time, resources, reducing workload or improve clinical pathways (R4,R11,R12,R13,R27,R35,R36,R39,R48,R50,R52,R53,R54,R55,R57,R61,R74,R91)
2. Implementation and changing practice
   1. How to support implementation (R5,R43,R49,R56,R78)
   2. Ensure buy-in from clinicians or physician cooperation (R40,R47)
   3. Change practice, i.e. adaptation and integration to real world existing workflows needed (R15, R57,R59,R62,R65,R71,R73,R75,R81,R88,R89)
3. Does the AI solution change clinical decision making?
   1. When does the AI solution propose an action? How, and who will actually implement it? (R21)
   2. Is staff’s approval needed for action proposed by the AI? (R21)
   3. Intended to replace physicians in a role they can already perform well or, alternatively, provide novel information that clinicians would not otherwise have (R37,R69)
   4. People displaced by these technologies or role expansion (R63)+reduce radiologist burnout (R53)
   5. Improve treatment choices (R54)
4. Multidisciplinary development required with active participation across all stakeholders (R24,R33,R40,R54,R56,R70,R73,R81,R83,R89)
5. Acceptability and algorithm trusted and easy to use
   1. Acceptance from users, e.g. patient and clinician acceptability (R5,R41,R88) or usability (R21,R47)
   2. Reassurance/trust when entrusting clinical care to machines, satisfaction, or convenience of patients and physician (R27,R41,R65) + algorithm is readily accessible and easy to use (R26,R72)
6. Extra workload
   1. Additional education or training needed to use AI solution, e.g. knowledge of statistics and data science (R21,R34,R35,R39, R40,R42,R43,R57,R71,R85,R89) + training opportunities in artificial intelligence technologies which improve the education of new physicians (R71,R74)
   2. Establish quality assurance (QA) program (R39) + During the initial stages of deployment, the output of the automatic segmentation software should be treated as if a trainee had performed the contouring − careful review is essential (R38) + Role of Chief Data Officer (CDO) in the hospital will be to safeguard the use of data for validation and training of machine learning systems and other data governance issues and ensure safety in integration into electronic health data and alignment with hospital’s policy (R63)
   3. Translating of technology into the healthcare setting is costly and time-consuming (R26) + time-consuming workflow (R78)

**2) How are outcomes (group 1 elements) measured, i.e. which specific outcome measures are mentioned?**

- Three types of AI in clinic: AI applications as alternative triage, replacement, or add-on in clinical workflows (R89)
- Radiologist burnout
- User time (e.g. time required for CC-Cruiser to make a diagnosis and treatment recommendation compared to the senior consultants)
- report comprehensiveness
- Usability, patient and clinician acceptability, reassurance/trust, satisfaction, convenience of patients and physician
- Potential for implementation in clinical practice (assessing the maturity), by means of classification in development phases (R78)
- Time used on education or training

**3) How many times is a topic or outcome group mentioned?**

- Changes in workflow (N=18)
- Implementation and changing practice (N=17)
- Change in care processes (N=6)
- Multidisciplinary development (N=10)
- Acceptability (N=10)
- Extra workload (N=17)

**4) Describe any overlap to other domains**

Topic 1 and 6 has implications for the economic domain.

**Summary of domain**

Benefits in the form of reductions in work flow and tasks related to imaging for the staff as a result of AI is mentioned frequently. So is the use of additional time related to implementation and training and the challenges related to ensuring acceptability. Regarding outcome measures, changes in time for the health care professionals and patient and clinician acceptability measures are mention.

| **Domain** | **Extractor** | **Reviewer** |
| --- | --- | --- |
| **8) Patients and Social** | **MGH** | **MNB** |

**1) Which overall effects, outcomes, value or impacts are mentioned - including future needs/challenges/topics relevant when evaluating the value of AI?**

1. **Patients’ willingness and comfortability**
2. Patients feel about the clinical benefits and tangible benefits for patients are more likely to engender trust and acceptance (R5,R56) + Patient acceptability/satisfaction (R41,R50,R67,R89,R88,R89,R91)
3. Avoiding compression and claustrophobia (R9)
4. Shortening scanning times, improvement of patients’ triage, privacy, comfort and motion artifacts (R22,R39,R77,R80,R83,R86)
5. Discrimination of patients with medical conditions by insurance system (R87)
6. Recognize and quantify high-level lifestyle activities of patients (R47)
7. Responding to participant requests for access to their data (R77)
8. Empower patient, change lifestyle, improve health, and sentiment data (R30)
9. **Technical aspects and reporting options in imaging process**
10. Automatically generation of multiple versions of the radiology report for different stakeholders (R53)
11. Determination of suitable label for an existing radiology report (R85)
12. **Clinical benefits and improved diagnosis**
13. Ensuring earlier diagnosis, tailored treatments, and more efficient follow-ups (R36,R85)
14. Better treatment outcome based on the improved clinical decision (R42,R56,R62,R74,R80)
15. More precise and personalized approach and minimizing the risk of misdiagnosis (R13,R82)
    1. Integration of patients' clinical characteristics (R44)
16. Stay healthy via continuous monitoring and coaching (R36)
17. Clinician acceptability and reliability (R41,R50,R56,R78,R86,R89,R88)
18. Choosing clinically relevant dataset to represent patients' group (R79)
19. **Challenges/benefits of AI integration to the healthcare system**
20. Performing studies using lifestyle and socioeconomic variables (R46) + socioeconomic aspects were inexistent (R45)
21. Ensuring patient safety standards by using “multidisciplinary AI team” (R73)
22. Capacity to accurately communicate neuroimaging results to remote participants (R77)
23. Return of research results and incidental findings to research participants (R77)
24. Mind-set shift in how patients entrust clinical care to machines (R88)

**2) How are outcomes (group 1 elements) measured, i.e. which specific outcome measures are mentioned?**

- Using online surveys for physicians’ opinions, participants’ feedback on their experience, the use of a cloud-based, multi-agent medical application for medical use in remote and rural areas (R5)
- Help patients make more informed activity choices (R47)
- Each generated version of report customized information and language designed for patients, families, oncologists, surgeons, and radiologists themselves (R53)
- Using satisfaction score for comparing satisfaction criteria (R67)
- Discrimination of patients with medical conditions based on predominantly genetic and not lifestyle-related (R87)
- Using time required for diagnosis and rating for overall satisfaction (R91)
- Socioeconomic variables (R46)

**3) How many times is a topic or outcome group mentioned?**

- Privacy of patients’ medical records: 3 times (R22,R77,R80)
- Ensuring earlier diagnosis, tailored treatments, and more efficient follow-ups: 2 times (R36,R85)
- Patient and clinician acceptability: 4 times (R41,R50,R56,R89)
- Better treatment outcome based on the improved clinical decision: 5 times (R42,R56,R62,R74,R80)
- Better satisfaction is using AI system: 3 times (R67,R88,R91)
- Being reliable when used for the assessment of unseen patients: 2 times (R78,R86)

**4) Describe any overlap to other domains**

There is an overlap between ethical domain and patients and social aspects as a great part of patients’ concerns belongs to privacy matter which could also count as ethical issue. Further, element 3 sentences has a clear overlap to the clinical domain. The topics of patient and clinician acceptability has overlap to the organisational domain.

**Summary of domain**

Patients, as the main customers of healthcare system, have an important role in integration of AI in healthcare systems. Studies address objects related to patients’ comfortability including easier imaging process and providing access to own data/report in a safe and secured platform. Further, better treatment outcome based on the improved clinical decision is the most discussed issue within published articles. Patients’ satisfaction as well as clinical benefits could result in better acceptability of AI technology in healthcare systems.

| **Domain** | **Extractor** | **Reviewer** |
| --- | --- | --- |
| **9) Legal** | **TK** | **MM** |

**1) Which overall effects, outcomes, value or impacts are mentioned - including future needs/challenges/topics relevant when evaluating the value of AI?**

1. GDPR and data privacy, security data ownership
   1. Security of data, data protection and GDPR safety (R5,R21,R22,R30,R31,R32,R35,R42,R51,R63,R65,R75,R76,R77,R86,R90)
   2. Who has access to data? (R5,R7,R21,R42,R63,R65,R66,R76)
   3. Who owns the data? (R22,R31,R42,R67,R90)
   4. Is data sufficiently anonymized? (R22,R35,R66,R75,R89)
   5. Can data be used to harm the patients? (R22)
   6. How will changes in data privacy rules affect the care they receive? (R22,R25,R66,R90)
2. Regulatory approval
   1. It is necessary before clinical implementation (R26,R31,R36,R42,R50,R54,R63,R82,R89)
   2. Who can approve the AI? (R31,R36,R54,R85)
   3. Continuous monitoring of third party AI companies to ensure compliance (R63,R89)
3. Responsibility for misdiagnosis
   1. Difference when AI is adjunct to clinician or when autonomous (R7,R31,R66,R73,R75,R76)

**2) How are outcomes (group 1 elements) measured, i.e. which specific outcome measures are mentioned?**

- AI-technologies must have regulatory approvals (R7,R25,R26,R31,R32,R75,R89,R90)
- Consent from patients to data collection (R32,R75,R90

**3) How many times is a topic or outcome group mentioned?**

- GDPR and data privacy (R7,R17,R21,R22,R25,R30,R31,R35,R42,R51,R63,R65,R66,R75,R76,R77,R82,R86,R89,R90)
- AI-technologies must have regulatory approvals (R26,R31,R32,R33,R50,R54,R63,R65,R69,R78,R82,R85,R89)
- Responsibility for misdiagnosis (R7,R31,R60,R66,R73,R75,R76,R82,R85,R86)

**4) Describe any overlap to other domains**

- Ethical (see topic 1)
- Patient (see topic 1e)
- Safety (see topic 3)

**Summary of domain**

The legal domain highlights three main themes decision makers must consider, when evaluating the legal aspects of AI-solutions. The first theme relates to *data security and privacy.* It is necessary to investigate to which degree the solution is compliant with regulations such as GDPR and considerations should be given to who has access to data, is data sufficiently anonymized and can the data be used to harm the patients. The second theme relates to *regulatory approvals*. It must be considered whether the AI-solution has been approved by FDA, EMA or other international or national regulatory agencies. The last theme relates to *responsibility for misdiagnosis.* It is important for decision makers to consider who holds the responsibility in case of misdiagnosis and also consider changes in responsibility when AI-solutions are autonomous compared to adjunct to clinical staff.

| **Domain** | **Extractor** | **Reviewer** |
| --- | --- | --- |
| **11) Other aspects** | **IF** | **MM** |

**1) Which overall effects, outcomes, value or impacts are mentioned - including future needs/challenges/topics relevant when evaluating the value of AI?**

- Overpromising language leaves studies vulnerable to being misinterpreted by the media and the public (R67)
- Offering the possibility of performing expensive and time-consuming screening programs in countries that otherwise cannot afford them (R86)

**Summary of domain**

Two issues were identified.
